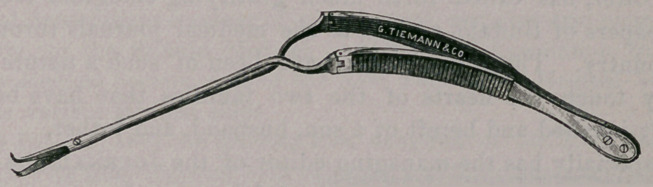# New Ear Scissors

**Published:** 1891-10

**Authors:** Alvin A. Hubbell

**Affiliations:** Buffalo, N. Y., Professor of Diseases of the Eye and Ear in the Medical Department of Niagara University; 212 Franklin Street


### NEW EAR SCISSORS.

By ALVIN A. HUBBELL, M. D., Buffalo, N. Y.,
Professor of Diseases of the Eye and Ear in the Medical Department of Niagara University.

In the treatment recently of a case of congenital occlusion of the external auditory canal, I found it necessary to use some instrument whose blades would cut one-sixth to one-fourth of an inch at right angles to the axis of the canal. The cutting points of Sexton's combination forceps, etc., were tried, but they did not fully answer the purpose. I presented the difficulties under which I was laboring to Messrs. George Tiemann & Co., with some suggestions as to what I needed, and they constructed the scissors represented in the accompanying cut.

The blades are bent near the point so as to extend about one-sixth of an inch on a curve nearly at right angles to their main portions. The latter are about half an inch long. The blades are so made and adjusted as to cut cleanly in both their axial and curved parts. The handles extend backward from the blade-joint an inch and a half, and are attached by joints to a forceps spring, which is bent downward like an ordinary ear forceps, so as to be out of the line of vision when being used. The instrument is light and yet strong, and is so ingeniously constructed as to open and close in the auditory canal with perfect ease, and at the same time without obstructing a view of the parts to be reached by it in a passage not below the medium size.

These scissors have proved eminently serviceable in cutting away growths and tissues from any part of the bottom of the auditory canal. They can be used much more easily and effectively than many other instruments, and will cut both lengthwise and transversely to the canal. They have appeared so useful to me that I venture to call the attention of the profession to them.

The instrument can be obtained of the manufacturers, George Tiemann & Co., 107 Park Row, New York.

212 Franklin Street, August 1, 1891.

## Figures and Tables

**Figure f1:**